# Carbon stock of the various carbon pools in Gerba-Dima moist Afromontane forest, South-western Ethiopia

**DOI:** 10.1186/s13021-019-0116-x

**Published:** 2019-02-02

**Authors:** Abyot Dibaba, Teshome Soromessa, Bikila Workineh

**Affiliations:** 1 College of Natural Sciences, Department of Biology, Debre Berhan University, P. O. Box 445, Debre Berhan, Ethiopia; 2 Center for Environmental Science, Addis Ababa University, P. O. Box No: 1176, Addis Ababa, Ethiopia; 3 College of Natural Sciences, Department of Plant Biology and Biodiversity Management, Addis Ababa University, P. O. Box 3434, Addis Ababa, Ethiopia

**Keywords:** Carbon, Forest, Gerba-Dima, Ethiopia

## Abstract

**Background:**

Unlike in the developed countries, Ethiopia does not have carbon inventories and databank to monitor and enhance carbon sequestration potential of different forests. Only small efforts have been made so far to assess the biomass and soil carbon sequestration at micro-level. This study was carried out to obtain sufficient information about the carbon stock potential of Gerba-Dima forest in south-western Ethiopia. A total of 90 sample plots were laid by employing stratified random sampling. Nested plots were used to collect data of the four carbon pools. For trees with a diameter range of 5 cm < diameter < 20 cm, the carbon stock was assessed from a plot size of 49 m^2^ (7 m * 7 m). For trees with a diameter range of 20 cm < diameter < 50 cm, the carbon stock was assessed from a plot size of 625 m^2^ (25 m * 25 m). For trees > 50 cm diameter, an additional larger sample of 35 * 35 m^2^ was used. Litter, herb and soil data were collected from 1 m^2^ subplot established at the center of each nested plot. To compute the above ground biomass carbon stock of trees and shrubs with DBH > 5 cm, their DBH and height were measured. The biomass carbon assessment of woody species having DBH < 5 cm, litter and herb were conducted by measuring their fresh weight in the field and dry weight in the laboratory.

**Results:**

The mean total carbon stock density of Gerba-Dima forest was found to be 508.9 tons carbon ha^−1^, out of which 243.8, 45.97, 0.03 and 219.1 tons carbon ha^−1^ were stored in the above ground biomass, below ground biomass, litter biomass and soil organic carbon, respectively.

**Conclusions:**

The existence of high carbon stock in the study forest shows the potential of the area for climate change mitigation. Thus, all stakeholders at the local and national level should work together to implement effective conservation measures and get benefit from the biocarbon fund.

**Electronic supplementary material:**

The online version of this article (10.1186/s13021-019-0116-x) contains supplementary material, which is available to authorized users.

## Background

The significance of forests in mitigating greenhouse gas emissions was recognized by the Kyoto Protocol. According to [1], forests and soils are potential sinks for elevated CO_2_ emissions and are being considered in the list of acceptable offsets. Sustainable forest development and forested landscape expansion are one of the fundamental approaches for reducing atmospheric carbon concentration. It is a safe, environmentally acceptable, and cost-effective way to capture and store large amounts of atmospheric carbon [1]. The simultaneous development of tradable carbon credits offers financial incentives for considering carbon storage in forest management decisions [2].

The tropical forests are said to play a major role in the global carbon cycle, storing up to about 46% of the world’s terrestrial carbon pool and about 11.55% of the world’s soil carbon pool, acting as a carbon reservoir and functioning as a constant sink of atmospheric carbon [3, 4]. A study carried out by Lugo and Brown showed that half of the presumed “matured forests” could also sequester carbon and the rate of sequestering carbon could be further improved if anthropogenic pressures are reduced or removed from these forests [5].

As sources of GHGs, deforestation represents about 20% of anthropogenic emissions [6, 7]. Although deforestation is reported to represent about 20% of the global GHGs emissions, regionally the figure varies. About 70% GHGs emissions is caused by deforestation in Africa [8]. For the entire world, carbon stocks in forest biomass reduced by an estimated 0.5 Gt annually during the period 2005–2010, mainly due to a reduction in the global forest area. On the other hand, the IPCC report estimated that the global forestry sector represents over 50% of the global greenhouse [9]. Consequently, forestry became the focus of global climate change policy and is given a key position in international climate treaties. While sustainable management, planting and rehabilitation of forests can conserve or increase forest carbon stocks, deforestation, degradation and poor forest management reduce them.

Ethiopia has one of the largest forest resources in the horn of Africa and presently existing data indicate that the forest resource of the country has a good potential in mitigating climate change. The forests resources of Ethiopia store 2.76 billion tons of carbon (about 10 billion tons of CO_2_) [10] in the aboveground biomass, which will be released to the atmosphere in 50 years if the deforestation continues at the present annual rate of about 2% [10].

Gerba Dima forest has been designated as Gerba Dima forest District by Oromia Forest Enterprise which is administered by regional government for the purpose of conserving the natural forest, wild life and expanding plantation forest for commercial purpose. This forest was also designated as part of the national forest priority area. The forest cover of South-western Ethiopia had declined from 38.4% in 1975 to 18.4% in 1996/97 [11]. Unlike the developed countries, Ethiopia does not have carbon inventories and databank to monitor and enhance carbon sequestration potential of different forests. Only small efforts have been made so far to assess the biomass and soil carbon sequestration at micro-level [12]. Despite the immense vegetation resource at Gerba Dima forest, no study has been conducted so far that aimed at investigating the carbon stock potential and associated dynamics of this forest. Thus, this study was carried out to obtain sufficient information about the carbon stock potential of Gerba-Dima forest in south-western Ethiopia, which could help as a reference for the conservation endeavour of the area and if carbon credit project will be implemented in the study forest.

## Materials and methods

### The study area

**Fig. 1:**
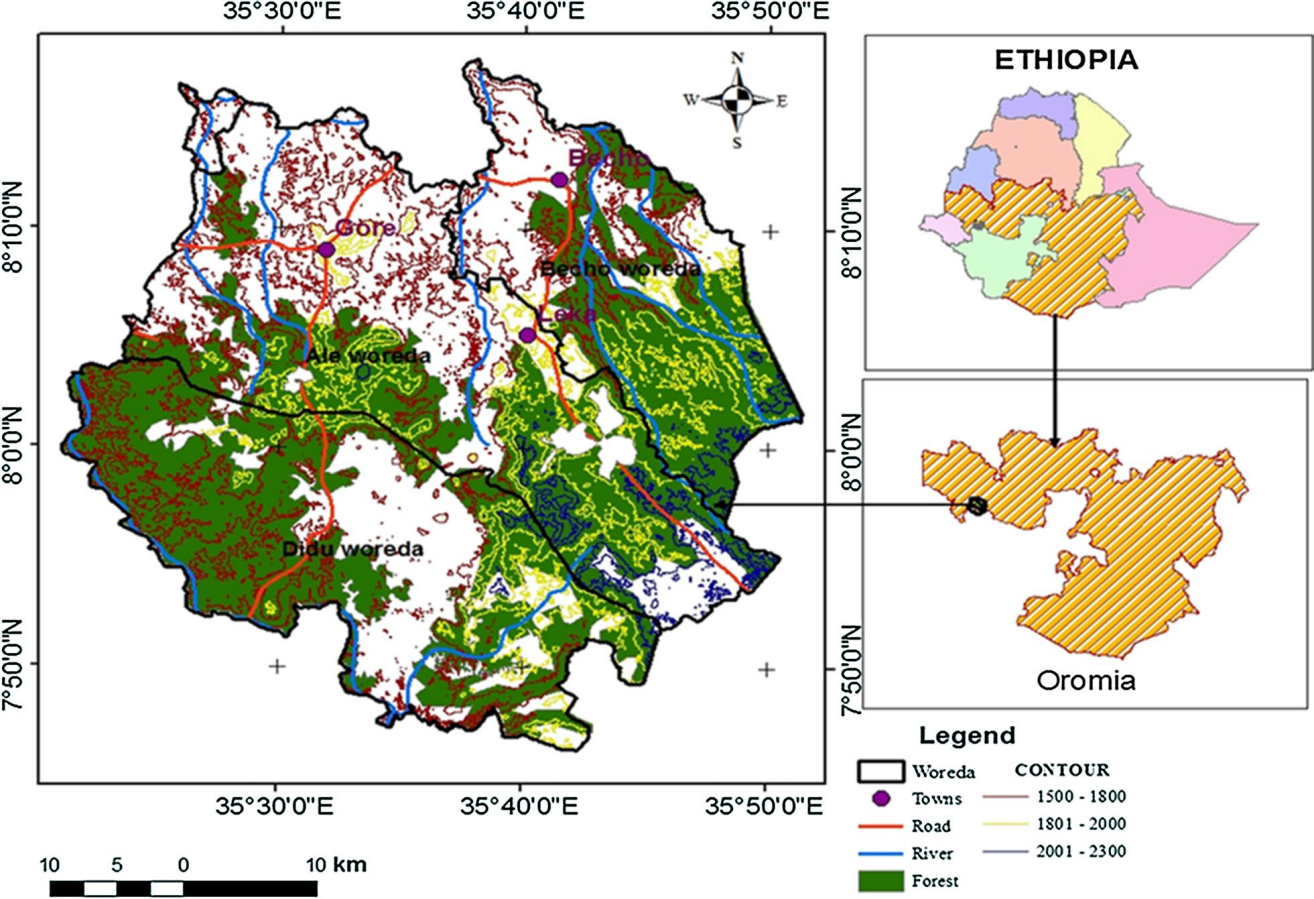
Map of the study area and sample sites

This study was conducted in Gerba-Dima located between 7°45′ to 8°10′North and 35°29’ to 35°50′East at about 630 km away from Addis Ababa and 30 km west of the zonal capital Metu. The study forest lies in Ale, Didu and Bacho districts of Illu Aba-Bora zone and forms part of the mountainous highlands west of the Great Rift Valley and is situated on undulating and dissected mountain ranges between 1582 m and 2285 m a.s.l. The forest area covers about 106,287.3 hectares (Fig. 1).

**Fig. 2:**
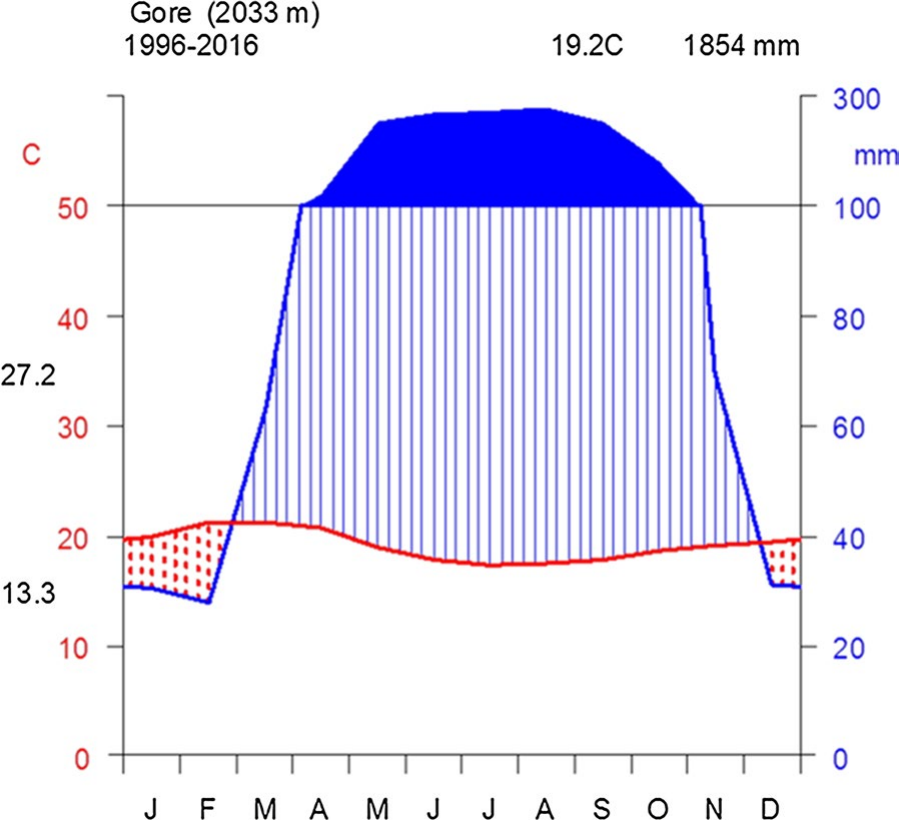
Climate diagram of Gore

The climate diagram showed unimodal rainfall pattern with monthly mean maximum and mean minimum temperature of 27.2 °C and 13.3 °C, respectively. The mean annual temperature was 19.2 °C. The mean annual rainfall of the study area was 1854 mm. The rainfall pattern showed low rainfall in December, January and February, gradually increasing to the peak period in August (Fig. 2).

The Underlying basement rock in the study area consists of intensively folded and faulted Precambrian rocks, overlain by Mesozoic marine strata and Tertiary basalt types [13]. The soils of the area are red or brownish ferrisols derived from the volcanic parent material. Other soil groups in the area include nitosols, acrisols, vertisols, and cambisols [14]. The vegetation type at Gerba Dima is part of the moist evergreen afromontane forest which is characterized by one or more closed strata of evergreen trees that may attain a height of 30 to 40 m [15]. The characteristic emergent species that form the upper canopy include *Pouteria adolfi*-*friederici. Albizia gummifera*, *A. schimperiana*, *A. grandibracteata*, *Sapium ellipticum*, *Euphorbia ampliphylla*, *Ekebergia capensis*, *Ficus sur*, *Hallea rubrostipulata*, *Ocotea kenyensis*, *Olea welwitschii*, *Polyscias fulva* and *Schefflera abyssinica* [15].

## Sampling design and measurements

In this study, a stratified random sampling design was used to collect carbon stock data of four carbon pools. Using Arc GIS version 10.3, the study area was stratified based on altitudinal gradient and three types of strata in the form of contour was established. Strata one was found at altitudinal range of 1500–1800 m while strata two and three were located between the altitudinal range of 1801–2000 m and 2001–2300 m respectively (Fig. 1). Sample plots were assigned in each stratum proportional to their area in the form of random points using Arc GIS. A total of 90 main sample plots along the contours were laid.

**Fig. 3:**
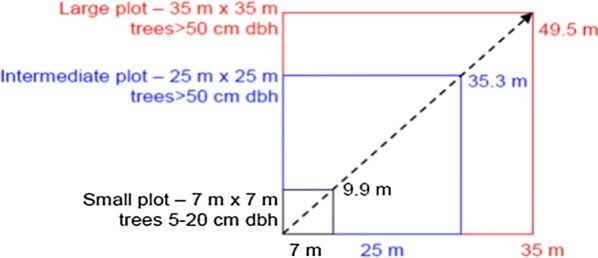
Nested plot design for sampling carbon pools

Nested plots were used as they are practical designs for sampling and recording discrete size classes of stems. The procedures were involved setting out three nested plots with 1225 m^2^ (35 m × 35 m) for trees above 50 cm diameter, 625 m^2^ (25 m × 25 m) for trees with a diameter range of 20 cm < diameter < 50 cm and 49 m^2^ (7 m × 7 m) for trees with a diameter of between 5 cm and 20 cm (Fig. 3). In each plot, diameter at breast height (1.3 cm) of all trees was measured using tree calliper while tree height was measured using clinometer. The plant species were determined by referring to published volumes of Flora of Ethiopia and Eritrea [16–21].

For leaf litter, one rectangular sub plot of 1 square meter in size was established at the center of each nested plot. The leaf litter within the 1 m^2^ sub plots were collected and weighed. A hundred grams of evenly mixed sub-samples were brought to the laboratory placing in a sample plastic bag to determine moisture content, from which total dry mass can then be calculated [22].

Within plots delineated for live trees, standing dead trees were also measured. The diameter at breast height (DBH) and decomposition state of the dead tree were recorded. For standing dead trees, with branches and twigs and resembles a live tree (except for leaves) were measured like trees. For the rest of standing dead trees at different stage of decomposition, the height of the trees, the diameter at ground level and at the top were measured [23]. Lying dead wood was sampled along 100 m line (using the line-intersect method) involving a lay out two lines of 50 m at right angles to determine biomass density [23]. Diameters and density classes were recorded and subsamples were collected to determine density in each of the three density classes (sound, intermediate, and rotten).

All herbaceous and other woody vegetation with DBH < 5 cm in diameter except coffee were cut into pieces and the fresh weight collected from 1 m^2^ were recorded following the same procedure with that of the litter [22, 23]. The soil samples for soil carbon determination were collected at the same sampling sub-quadrats recommended for litter sampling. From the center of each plot and/or sub-plot a pit of up to 30 cm in depth was dug to best represent forest types in terms of slope, aspect, vegetation, density, and cover [23]. A hundred grams of composite sample was collected from one plot by digging the soil with the help of standardized 300 cm^3^ metal soil sampling corer. The soil samples collected from plot were brought to the laboratory placing in a sample paper bags. Then, the bulk density and amounts of soil organic matter were determined.

## Estimation of biomass and carbon stock

From the different available allometric equations to estimate the above ground biomass, the model that was developed by [24] is recommendable for the study site since the general criteria described by the author can agree with the study area primarily due to similarity of life zone. Equations that incorporate more than one tree parameters improve the reliability of forest biomass estimation [25].

$${\text{AGB}}\;est = \rho *d2*H*0.0559$$

where AGB est = above ground biomass (kg), d = DBH (cm), H = height (m), and ρ = basic wood density (g cm^−3^).

Specific wood densities for woody species were acquired from Global Wood Density Database [26, 27].

To estimate below-ground biomass, the equation developed by [28] was used.

$${\text{BGB est }} = {\text{ AGB}} \times \, 0. 2$$

where, BGB est is below ground biomass, AGB is above ground biomass, 0.2 is conversion factor (or 20% of AGB).

Estimation of the amount of biomass in the leaf litter was calculated following [23].

$$LB = \frac{Wfield}{A} \times \left[ {\frac{{{\text{Wsub}}\_{\text{sample}}\left( {\text{dry}} \right)}}{{{\text{Wsub}}\_{\text{sample}}\left( {\text{fresh}} \right)}}} \right] \times \frac{1}{10,000}$$

where LB = Litter (biomass of litter t ha^−1^); W field = weight of wet field sample of litter sampled within an area of size 1 m^2^ (g); A = size of the area in which litter were collected (ha).

W sub-sample, dry = weight of the oven-dry sub-sample of litter taken to the laboratory to determine moisture content (g), and W sub-sample, fresh = weight of the fresh sub-sample of litter taken to the laboratory to determine moisture content (g).

Carbon stocks in litter biomass was calculated

$${\text{CL }} = {\text{ LB }} \times \, \% {\text{ C}}$$

where, CL is total carbon stocks in the litter in t ha^−1^, % C is carbon fraction determined in the laboratory.

Estimation of herbaceous biomass and carbon stock was conducted following the same procedure with that of the litter [23].

Estimation of the amount of biomass in the Non Tree Woody Species (NTWS) with DBH < 5 cm was calculated following [23].

$$NTWSB = \frac{Wfield}{A} \times \left[ {\frac{{{\text{Wsub}}\_{\text{sample}}\left( {\text{dry}} \right)}}{{{\text{Wsub}}\_{\text{sample}}\left( {\text{fresh}} \right)}}} \right] \times \frac{1}{10,000}$$

where NTWSB = Non tree Woody Species biomass in t ha^−1^. W field = weight of wet field sample of NTWS sampled within an area of size 1 m^2^ (g); A = size of the area in which NTWS were collected (ha); W sub-sample, dry = weight of the oven-dry sub-sample of NTWS taken to the laboratory to determine moisture content (g), and W sub-sample, fresh = weight of the fresh sub-sample of NTWS taken to the laboratory to determine moisture content (g).

The carbon content in NTWS is calculated by multiplying the biomass with the [29] default carbon fraction of 0.47.

For standing dead wood (SDW) which has branches, the biomass was estimated using the allometric equation selected for estimation of above ground biomass [23].

For the rest of standing dead wood, the biomass was estimated using wood density and volume calculated from truncated cone [23].

$${\text{Volume }}\left( {{\text{m}}^{ 3} } \right) \, = 1/3\;\pi {\text{ h }}\left( {{\text{r}}_{ 1}^{ 2} + {\text{ r}}_{ 2}^{ 2} + {\text{ r}}_{ 1} {\text{x r}}_{ 2} } \right)$$

where h = the height in meters, r_1_ = the radius at the base of the tree, r_2_ = the radius at the top of the tree.

$${\text{Biomass }} = {\text{ Volume }} \times {\text{ Wood density }}\left( {{\text{from samples}}} \right)$$

The biomass of lying dead wood was estimated by the equation given below [23]:

$$LDW = \mathop \sum \limits_{i = 0}^{n} V \times s$$

where LDW = lying dead wood, V = volume and s = specific density of each density class.

The volume of lying dead wood per unit area is estimated by:

$${\text{V }} = \, \pi^{ 2} \left( {{\text{D}}^{ 2} / 8 {\text{L}}} \right)$$

where V is the volume in m^3^/ha; D is diameter of the dead wood tree and L is the length of the line transect.

The carbon content in dead wood is calculated by multiplying total biomass of dead wood with the [29] default carbon fraction of 0.47.

Soil organic carbon was computed using the formula [23]

$${\text{SOC }} = {\text{ BD }}*{\text{ D }}* \, \% {\text{C}}$$

where SOC = soil organic carbon stock per unit area (t ha^−1^), BD = soil bulk density (g cm^−3^), D = the total depth at which the sample was taken (30 cm), and  %C = Carbon concentration (%).

For percentage of carbon determination, the loss on ignition (LOI) method was used [30]. In this method; initially fresh weight of samples were taken on the field, and then dried at 65 °C in the oven for 48 h to take dry weight. Oven dried grind samples were taken (5.00 g) in pre-weighted crucibles, after that put in the furnace at 550 °C for 1 h to ignite. The crucibles were cooled slowly inside the furnace. After cooling, the crucibles with ash were weighed and percentage of organic carbon was calculated following [30].

$$\% Ash = \left[ {\frac{{{\text{W}}3 - {\text{W}}1}}{{{\text{W}}2 - {\text{W}}1}}} \right] \times 100$$

$$\% C = \left( {100 - \% Ash} \right) \times 0.58$$

By considering 58% Carbon in ash-free soil material. where W1—Weight of crucible, W2—Weight of the oven-dried grind sample and crucible, and W3—Weight of ash and crucible.

The total carbon stock density is calculated by summing the carbon stock densities of the individual carbon pools [23].

$${\text{C}}_{\text{density}} = {\text{ C}}_{\text{AGB}} + {\text{ C}}_{\text{BGB}} + {\text{ C}}_{\text{L}} + {\text{ C}}_{\text{DW}} + {\text{SOC}}$$

where C _density_ = Carbon stock density for all pools [t ha^−1^]

$${\text{C}}_{\text{AGTB}} = {\text{ Carbon in above}} - {\text{ground tree biomass }}\left[ {{\text{t C ha}}^{ - 1} } \right];$$

C_BGB_ = Carbon in below-ground biomass [t C ha^−1^]; C _L_ = Carbon in dead litter [t C ha^−1^]; C_DW_ = Carbon in dead wood; SOC = Soil organic carbon

Physiographical variables, namely altitude, geographic coordinates, slope and aspect, were recorded for each quadrat using GPS, Clinometer and Compass respectively. These soil samples were analyzed for texture on the basis of Bouycous Hydrometer method. Species diversity was calculated using Shannon diversity index (H′). The various carbon pools were correlated with one another and with aforementioned environmental variables.

## Results

### Biomass and carbon stock estimation of various carbon pools

The density of woody species in the study area was 1829 individuals per hectare. The mean, minimum and maximum DBH of trees were 27.6 cm, 5 cm and 490 cm respectively. The minimum and maximum DBH was exhibited by *Maytenus gracilipes* and *Schefflera abyssinica* respectively. The mean, minimum and maximum tree heights were 12 m, 2 m and 50 m respectively. The minimum and maximum heights were exhibited by *Vepris dainellii* and *Pouteria adolfi*-*friederici* respectively.

The mean above ground carbon stock in the study site was 243.85 ± 17.27 t ha^−1^. The first top ten species which stored the highest above ground carbon stock of the forest were *Ekebergia capensis*, *Schefflera abyssinica*, *Pouteria adolfi*-*friederici*, *Prunus africana*, *Elaeodendron buchananii*, *Olea welwitschii*, *Sapium ellipticum*, *Trilepisium madagascariense*, *Polyscias fulva*, *Ficus sur* with values of 89.75, 40.30, 29.62, 21.03, 20.12, 16.31, 16.21, 13.43, 10.14, 9.93 t ha^−1^ respectively. The least above ground carbon stock in the forest was recorded by species of *Clausena anisata*, *Psychotria orophila*, *Pterolobium stellatum*, *Flacourtia indica*, *Solanaceo manni*, *Phyllanthus sepialis*, *Coffea arabica*, *Deinbollia kilimandscharica*, *Solanecio gigas* and *Vernonia rueppellii* with values of 0.52, 0.47, 0.45, 0.44, 0.42, 0.34, 0.32, 0.26, 0.25, 0.17 t ha^−1^ respectively.

The mean below ground carbon stock of the study site was 45.97 ± 3.46 t ha^−1^. Mean total carbon stock of litter in the study site was 0.026 ± 0.005 t ha^−1^. Mean total carbon stock of herb layer of the study site was 0.007 ± 0.0004 t ha^−1^. The mean NTWS (non tree woody species with DBH < 5 cm) carbon stock of the study site was 0.12 ± 0.01 t ha^−1^. The mean SDW carbon stock of the study site was 1.83 ± 0.55 t ha^−1^. The mean LDWC stock in the study area was 2.81 ± 0.35 t ha^−1^.

**Fig. 4:**
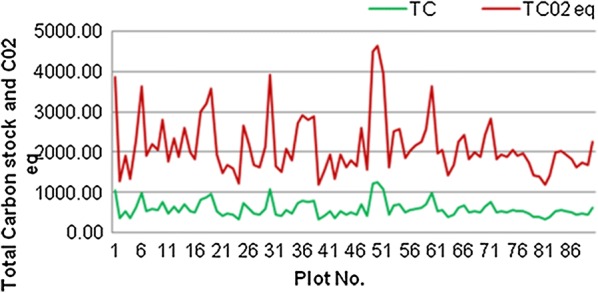
Total carbon stock (TC) and CO_2_eq. of each plot

The soil bulk density ranged from 0.4 g cm^−3^ to 0.9 g cm^−3^. On the other hand, 0.58 g cm^−3^ was the average soil bulk density indicating the presence of high soil organic matter in mineral soils. The largest soil organic matter was 34.91% whereas 10.15% is the lowest value. The carbon content of soil carbon pool ranged from minimum storage of 106.68 t ha^−1^ to a maximum of 279.45 t ha^−1^ per plot of the study site. The mean soil carbon stock of the study area was 162.62 ± 3.20 t ha^−1^. The total carbon stock values of the study forest ranged from a minimum of 212.61 in the region of plot 39 to a maximum of 1155 t ha^−1^ in the region of plot 50 (Fig. 4). The mean carbon stock in all carbon pool of the study site was 457.22 ± 20.59 t ha^−1^.

### Comparison and correlation between different carbon pools

Carbon stock in a different pool of the study site shows variation. The highest percentage of carbon was stored in soil organic carbon pool (49%) followed by the above ground carbon pool (41%), below ground carbon pool (9%) and dead wood carbon pool (1%) respectively. Compared with aforementioned carbon pool, the contribution of herbaceous, litter and non-tree woody vegetation carbon pools were insignificant.

Correlation of the various carbon pools with one another were tested using Pearson’s product moment correlation coefficient (Table 1). The statistically strong positive correlation was observed between NTWSC and Carbon stock in litter, SOC and Carbon stock in Herb, SOC and NTWSC.

**Table 1:** Pearson’s product moment correlations coefficient and P value between Carbon pools

	AGC ton/Ha	Carbon stock in litter ton/ha	Carbon stock in Herb ton/ha	Carbon stock in NTWS ton/ha	DWC ton/ha	SOC ton/ha
AGC ton/ha	1					
	0.000					
Carbon stock in litter ton/ha	0.032	1				
	0.761					
Carbon stock in Herb ton/ha	0.171	0.121	1			
	0.107	0.255				
Carbon stock in NTWS ton/ha	0.129	0.249*	0.119	1		
	0.226	0.018	0.264			
DWC ton/ha	− 0.121	0.036	− 0.006	− 0.016	1	
	0.257	0.736	0.954	0.878		
SOC ton/ha	− 0.082	0.124	0.265*	0.230*	− 0.017	1
	0.440	0.242	0.011	0.029	0.871	

Cell Contents: Pearson correlation (upper cell) and P Value (lower cell)

**P < 0.01, *P < 0.05

Relationship between carbon stocks and environmental gradients correlation of the various carbon pools with eight environmental variables were tested using Pearson’s product-moment correlation coefficient (Table 2) Statistically strong positive correlation was observed between AGC and diversity, BGC and diversity, carbon stock in litter and clay, carbon stock in herb and Altitude, DWC (dead wood carbon) and Altitude, DWC and clay, SOC and altitude, SOC and sand. On the other hand strongly statistically negative correlation was shown between AGC and disturbance, BGC and disturbance, Carbon stock in litter and Sand, NTWVC and disturbance, DWC and disturbance, SOC and clay.

**Table 2:** Pearson’s product moment correlations coefficient and P value between Carbon pools and environmental gradients

	Slope	Aspect	Disturbance	Altitude	SAND	CLAY	SILT	Diversity
AGC	0.030	− 0.055	− 0.236*	0.141	− 0.097	0.093	0.011	0.207*
	0.782	0.607	0.025	0.184	0.362	0.384	0.917	0.050
	0.798	0.647	0.034	0.311	0.746	0.793	0.888	0.017
Carbon stock in litter	− 0.038	− 0.162	− 0.189	0.120	− 0.295**	0.224*	0.160	0.002
	0.722	0.128	0.074	0.259	0.005	0.034	0.133	0.983
Carbon stock in Herb ton/ha	0.050	− 0.001	− 0.191	0.368**	− 0.160	0.105	0.121	0.131
	0.642	0.989	0.071	0.000	0.133	0.323	0.255	0.218
Carbon stock in NTWV ton/ha	− 0.027	0.023	− 0.239*	− 0.043	− 0.076	0.035	0.089	0.046
	0.797	0.827	0.023	0.690	0.479	0.742	0.404	0.665
DWC	− 0.065	− 0.172	− 0.018	0.290**	− 0.234*	0.212*	0.054	0.028
	0.540	0.106	0.868	0.006	0.026	0.045	0.616	0.790
SOC	0.111	0.188	− 0.014	0.236*	0.260*	− 0.220*	− 0.092	0.015
	0.299	0.077	0.896	0.025	0.013	0.037	0.389	0.887

Cell Contents: Pearson correlation (upper cell) and P Value (lower cell)

**P < 0.01, * P < 0.05

## Discussion

### Carbon stock of Gerba-Dima forest

Species which stored the highest carbon stock were the dominant species exhibiting higher basal area in the study forest. Thus, the plant species represented by individuals with larger DBH have a significant contribution to the carbon storage in this forest and their removal significantly alters the biomass dynamics of the forests. Bigger trees with higher diameter store the largest stocks of carbon within biomass and are often impacted by forest degradation and deforestation [8]. The AGC and BGC in Gerba-Dima forest were higher than values reported by IPCC [29, 31] for tropical forests. The higher average carbon stock in above ground biomass in the study site could be related to the higher tree height, DBH and basal area in the forest. Tree species like *Pouteria adolfi*-*friederici* reached as tall as 50 m and the basal area of this forest was 65.05 m^2^/ha which is higher than the normal basal area value for virgin tropical forests in Africa (23–37 m^2^/ha) [32]. The mean litter carbon stock in Gerba-Dima forest was low. Since the study area is located in tropical areas, the rate of decomposition is relatively fast [33]. Hence, the lowest carbon stock in litter pool could probably be due to the high rate of litter decomposition.

The mean carbon stock of herb layer in Gerba-Dima forest was very low compared with the herbaceous mean carbon stock of tropical forest in Eastern Panama (0.11 t ha^−1^) [34]. The decrease in the amount of carbon stock in herbaceous layer of the study forest may be attributed to the shading effect of the canopy trees which reduce light penetration and can also affect physical and chemical soil properties for the growth of herb and grasses [35].

The mean SOC of Gerba Dima forest was higher than mean SOC of Tropical & Subtropical Moist Broadleaf forests (57 t ha^−1^) [36]. The variation of SOC between different vegetation types could be attributed to the presence of different tree species, soil nutrient availability, climate, topography, disturbance regime, the number of soil profiles considered, the soil layer that are considered and method employed to detect the amount of SOC [36, 37]. The amount of organic matter and soil carbon stock is an outcome of the balance between inputs (mostly from biomass detritus) and outputs to the system (mostly decomposition and transport), which are driven by diverse parameters of natural or human origins [38].

The mean carbon stock in all carbon pool of the study site was higher than the average value of tropical forests. According to [29], biome-average tropical forest carbon stock estimates of the sub-Saharan Africa tropical equatorial forest, tropical seasonal forest and tropical dry forest are 200, 152 and 72 t ha^−1^ respectively. The variation in carbon stock between different forest types could be attributed to imprecise measurements of tree variables, inefficiency of allometric models, the presence of bigger sized trees with a higher basal area, a higher density of woody species and anthropogenic disturbance [39].

Statistically significant positive correlation between NTWSC and SOC as well as herbaceous carbon stock and SOC can be explained with the fact that herbs and NTWSC are either annuals or short-lived perennials which die and mixed with soil frequently to enrich the SOC pool [40]. The positive significant correlation between AGC and diversity could be attributed to the fact that more diverse plant communities have a higher chance of including highly productive species that dominates the community [41, 42].

The result of this study revealed that AGC, BGC and NTWVC were negatively correlated with disturbance and such pattern of correlation clearly confirmed that forest disturbance reduced the capacity of the forests to sequester carbon. Disturbances also alter the forest productivity, may release C directly into the atmosphere and transfer large amounts of C from biomass into detritus, soils or forest products [43]. With increasing disturbance frequency, a greater proportion of the forest is found in younger age classes. Young and immature stands in the landscape contain less C than mature stands [44].

In Gerba-Dima forest, SOC and DWC were positively correlated with altitude. The effect of elevation was complex and was probably indirect. Generally, temperature decreased and precipitation increased with increasing altitude. The changes in climate along altitudinal gradients influence the composition and productivity of vegetation and, consequently, affect the quantity and turnover of SOM [45]. The decline in temperature accompanied with an increase in elevation could reduce SOC and DWC turnover rates, leading to increases in SOC and DWC levels [46]. Generally, SOC is significantly higher in areas where the precipitation is greater. Higher precipitation is generally associated with higher rates of vegetation growth, and thus, with higher rates of organic carbon input and SOC accumulation.

## Conclusion

The study of carbon stock of the various carbon pools in Gerba-Dima forest revealed the existence of high carbon stock which was very high compared with the average value of tropical forests. From the different carbon pools, AGC was the highest in the study forest. However, the contribution of herbaceous, litter and non-tree woody vegetation carbon pools were insignificant. The various carbon pools showed a significant correlation to different environmental variables. AGC pool was positively correlated with species diversity while disturbance negatively affects the carbon stock of these pools. Positive significant correlation was also observed between SOC and altitude. The existence of high carbon stock in the study forest shows the potential of the area for climate change mitigation. Thus, all stakeholders at local and national level should work together to implement effective conservation measures and get benefit from biocarbon fund.

## Additional files

**Additional file 1.** Carbon stock of all carbon pools for each study plot in Gerba Dima forest.

**Additional file 2: Appendix S1.** AGB, BGB, AGC, BGC and Carbon sequestered (CO_2_ equivalent) per tree of species in Gerba Dima Forest.

**Additional file 3.** Bulk Density, SOM, SOC and CO2 equivalent for each study plot in Gerba Dima Forest.

## Abbreviations

AGB: above ground biomass
AGC: above ground carbon
BGB: below ground biomass
BGC: below ground carbon
DBH: diameter at breast height
DWC: dead wood carbon
NTWSC: non tree woody species carbon
SOC: soil organic carbon
