# Supplementary material for: Carbon stock of the various carbon pools in Gerba-Dima moist Afromontane forest, South-western Ethiopia
Source: Carbon Balance Manag. 2019 Feb 2;14:1. doi: 10.1186/s13021-019-0116-x (PMC6446976; doi:10.1186/s13021-019-0116-x)
Supplement: Supplementary file 1 — Additional file 1. Carbon stock of all carbon pools for each study plot in Gerba Dima forest. [file 13021_2019_116_MOESM1_ESM.docx]

| **Plot no** | **AGC ton ha^-1^** | **BGC ton ha^-1^** | **Litter C (tha-1** | **Herb C (t ha-1)** | **NTWSC (t ha-1)** | **SOC (t ha-1)** | **DWC (t ha-1)** | **Total C stock (t ha-1)** |
| --- | --- | --- | --- | --- | --- | --- | --- | --- |
| Plot 1 | 700.11 | 122.7 | 0.02 | 0.006 | 0.13 | 130.764 | 4.23 | 957.96 |
| Plot 2 | 84.71 | 10.97 | 0.02 | 0.005 | 0.03 | 137.862 | 14.86 | 248.46 |
| Plot 3 | 236.56 | 40.5 | 0.03 | 0.005 | 0.06 | 135.582 | 0.90 | 413.64 |
| Plot 4 | 147.31 | 12.46 | 0.01 | 0.012 | 0.04 | 118.389 | 0.94 | 279.16 |
| Plot 5 | 233.99 | 35.34 | 0.02 | 0.012 | 0.08 | 203.184 | 3.39 | 476.02 |
| Plot 6 | 550.34 | 103.21 | 0.02 | 0.021 | 0.09 | 191.94 | 2.89 | 848.51 |
| Plot 7 | 191.49 | 18.7 | 0.04 | 0.006 | 0.06 | 173.376 | 2.10 | 385.77 |
| Plot 8 | 240.9 | 30.63 | 0.45 | 0.008 | 0.32 | 188.478 | 6.12 | 466.91 |
| Plot 9 | 266.64 | 46.95 | 0.02 | 0.009 | 0.10 | 134.568 | 1.22 | 449.51 |
| Plot 10 | 395.94 | 52.23 | 0.04 | 0.004 | 0.07 | 169.344 | 12.76 | 630.39 |
| Plot 11 | 105.8 | 9.3 | 0.02 | 0.005 | 0.14 | 202.665 | 4.08 | 322.01 |
| Plot 12 | 315.28 | 32.08 | 0.04 | 0.004 | 0.04 | 158.946 | 4.25 | 510.64 |
| Plot 13 | 176.84 | 23.77 | 0.04 | 0.010 | 0.13 | 167.973 | 9.61 | 378.37 |
| Plot 14 | 285.24 | 29.29 | 0.05 | 0.013 | 0.12 | 222.72 | 3.76 | 541.18 |
| Plot 15 | 216.71 | 16.91 | 0.06 | 0.004 | 0.13 | 171.867 | 1.39 | 407.07 |
| Plot 16 | 203.45 | 32.91 | 0.02 | 0.015 | 0.07 | 147.072 | 2.85 | 386.40 |
| Plot 17 | 422.69 | 84.54 | 0.04 | 0.010 | 0.02 | 170.868 | 1.17 | 679.33 |
| Plot 18 | 377.79 | 75.56 | 0.07 | 0.014 | 0.25 | 234.918 | 2.65 | 691.26 |
| Plot 19 | 496.23 | 99.25 | 0.06 | 0.019 | 0.13 | 217.602 | 0.76 | 814.06 |
| Plot 20 | 175.78 | 35.16 | 0.05 | 0.006 | 0.23 | 175.053 | 0.87 | 387.15 |
| Plot 21 | 93.61 | 18.72 | 0.02 | 0.007 | 0.09 | 156.426 | 12.79 | 281.66 |
| Plot 22 | 119.72 | 23.94 | 0.02 | 0.007 | 0.14 | 153.384 | 37.87 | 335.09 |
| Plot 23 | 105.7 | 21.14 | 0.03 | 0.011 | 0.17 | 158.364 | 17.29 | 302.70 |
| Plot 24 | 76.9 | 15.38 | 0.01 | 0.005 | 0.09 | 128.949 | 3.24 | 224.58 |
| Plot 25 | 350.05 | 70.01 | 0.03 | 0.007 | 0.09 | 156.06 | 17.96 | 594.20 |
| Plot 26 | 169.79 | 33.96 | 0.03 | 0.006 | 0.17 | 228.375 | 11.67 | 444.00 |
| Plot 27 | 89.14 | 17.83 | 0.02 | 0.005 | 0.04 | 162.3 | 49.61 | 318.95 |
| Plot 28 | 119.3 | 23.86 | 0.02 | 0.006 | 0.17 | 157.818 | 13.90 | 315.08 |
| Plot 29 | 240.47 | 48.09 | 0.02 | 0.005 | 0.09 | 157.464 | 3.21 | 449.35 |
| Plot 30 | 666.04 | 133.21 | 0.02 | 0.007 | 0.17 | 146.889 | 7.45 | 953.79 |
| Plot 31 | 185.63 | 37.13 | 0.02 | 0.008 | 0.14 | 115.056 | 11.45 | 349.43 |
| Plot 32 | 121.7 | 24.34 | 0.02 | 0.007 | 0.17 | 148.608 | 8.13 | 302.98 |
| Plot 33 | 171.87 | 34.37 | 0.02 | 0.013 | 0.08 | 207.708 | 9.31 | 423.37 |
| Plot 34 | 163.51 | 32.7 | 0.02 | 0.012 | 0.17 | 152.586 | 1.85 | 350.85 |
| Plot 35 | 376.88 | 75.38 | 0.02 | 0.005 | 0.09 | 148.905 | 2.03 | 603.31 |
| Plot 36 | 400.6 | 80.12 | 0.03 | 0.011 | 0.32 | 176.292 | 1.50 | 658.87 |
| Plot 37 | 378.68 | 75.74 | 0.02 | 0.007 | 0.06 | 152.28 | 12.21 | 618.99 |
| Plot 38 | 367.57 | 73.51 | 0.02 | 0.007 | 0.32 | 188.1 | 0.99 | 630.52 |
| Plot 39 | 57.76 | 11.55 | 0.02 | 0.007 | 0.10 | 142.614 | 0.56 | 212.61 |
| Plot 40 | 134.43 | 26.89 | 0.03 | 0.005 | 0.32 | 135.387 | 3.12 | 300.18 |
| Plot 41 | 194.54 | 38.91 | 0.03 | 0.003 | 0.07 | 165.822 | 0.52 | 399.90 |
| Plot 42 | 91.18 | 18.24 | 0.05 | 0.004 | 0.14 | 134.55 | 1.85 | 246.01 |
| Plot 43 | 209.71 | 41.94 | 0.02 | 0.002 | 0.04 | 159.138 | 0.76 | 411.61 |
| Plot 44 | 146.13 | 29.23 | 0.01 | 0.005 | 0.13 | 133.245 | 4.52 | 313.27 |
| Plot 45 | 222.76 | 44.55 | 0.02 | 0.005 | 0.12 | 120.624 | 0.34 | 388.42 |
| Plot 46 | 160.98 | 32.2 | 0.02 | 0.011 | 0.08 | 142.23 | 2.35 | 337.87 |
| Plot 47 | 365.92 | 73.18 | 0.02 | 0.011 | 0.03 | 148.008 | 2.06 | 589.23 |
| Plot 48 | 128.51 | 25.7 | 0.02 | 0.010 | 0.03 | 153.09 | 0.52 | 307.88 |
| Plot 49 | 770.19 | 154.04 | 0.01 | 0.007 | 0.05 | 170.88 | 1.66 | 1096.84 |
| Plot 50 | 872.25 | 174.45 | 0.02 | 0.006 | 0.13 | 106.68 | 1.39 | 1154.92 |
| Plot 51 | 652.29 | 130.46 | 0.03 | 0.005 | 0.07 | 164.775 | 6.36 | 953.99 |
| Plot 52 | 169.69 | 33.94 | 0.02 | 0.010 | 0.02 | 131.718 | 0.64 | 336.04 |
| Plot 53 | 308.49 | 61.7 | 0.01 | 0.010 | 0.25 | 169.92 | 7.45 | 547.83 |
| Plot 54 | 352.21 | 70.44 | 0.02 | 0.008 | 0.32 | 152.04 | 1.61 | 576.65 |
| Plot 55 | 213.92 | 42.78 | 0.01 | 0.006 | 0.13 | 138.726 | 6.69 | 402.27 |
| Plot 56 | 270.17 | 54.03 | 0.01 | 0.005 | 0.23 | 116.1 | 2.46 | 443.01 |
| Plot 57 | 159.82 | 31.96 | 0.01 | 0.008 | 0.32 | 241.38 | 3.38 | 436.88 |
| Plot 58 | 247.63 | 49.53 | 0.01 | 0.004 | 0.14 | 184.59 | 1.18 | 483.09 |
| Plot 59 | 280.03 | 56.01 | 0.01 | 0.005 | 0.17 | 212.574 | 2.64 | 551.43 |
| Plot 60 | 627.73 | 125.55 | 0.01 | 0.002 | 0.09 | 124.785 | 0.14 | 878.31 |
| Plot 61 | 174.9 | 34.98 | 0.01 | 0.006 | 0.09 | 177.654 | 6.89 | 394.53 |
| Plot 62 | 213.62 | 42.72 | 0.02 | 0.007 | 0.32 | 166.692 | 1.66 | 425.04 |
| Plot 63 | 67.3 | 13.46 | 0.01 | 0.003 | 0.04 | 170.52 | 1.78 | 253.11 |
| Plot 64 | 108.59 | 21.72 | 0.01 | 0.005 | 0.17 | 172.725 | 0.50 | 303.72 |
| Plot 65 | 252.96 | 50.59 | 0.01 | 0.005 | 0.09 | 168.795 | 0.50 | 472.95 |
| Plot 66 | 104.61 | 20.92 | 0.02 | 0.008 | 0.32 | 279.45 | 0.32 | 405.65 |
| Plot 67 | 220.42 | 44.08 | 0.01 | 0.005 | 0.14 | 126.06 | 0.43 | 391.14 |
| Plot 68 | 219.04 | 43.81 | 0.01 | 0.005 | 0.32 | 148.8 | 2.63 | 414.62 |
| Plot 69 | 161.66 | 32.33 | 0.01 | 0.007 | 0.08 | 174.522 | 4.30 | 372.91 |
| Plot 70 | 325.29 | 65.06 | 0.01 | 0.005 | 0.32 | 141.414 | 1.28 | 533.38 |
| Plot 71 | 429.25 | 85.85 | 0.01 | 0.007 | 0.32 | 132.048 | 5.38 | 652.87 |
| Plot 72 | 196.57 | 39.31 | 0.02 | 0.003 | 0.02 | 139.995 | 2.47 | 378.38 |
| Plot 73 | 224.55 | 44.91 | 0.01 | 0.005 | 0.03 | 142.158 | 0.85 | 412.51 |
| Plot 74 | 220.91 | 44.18 | 0.01 | 0.005 | 0.04 | 140.184 | 3.49 | 408.82 |
| Plot 75 | 226.25 | 45.25 | 0.01 | 0.006 | 0.05 | 162.9 | 1.09 | 435.56 |
| Plot 76 | 163.79 | 32.76 | 0.01 | 0.004 | 0.02 | 181.908 | 1.23 | 379.72 |
| Plot 77 | 169.85 | 33.97 | 0.01 | 0.005 | 0.04 | 185.256 | 2.69 | 391.82 |
| Plot 78 | 130.09 | 26.02 | 0.02 | 0.005 | 0.03 | 171.072 | 1.57 | 328.80 |
| Plot 79 | 80.32 | 16.06 | 0.02 | 0.006 | 0.02 | 157.092 | 2.85 | 256.36 |
| Plot 80 | 79.16 | 15.83 | 0.02 | 0.006 | 0.01 | 152.88 | 1.07 | 248.98 |
| Plot 81 | 77.03 | 15.41 | 0.01 | 0.005 | 0.04 | 121.824 | 0.48 | 214.80 |
| Plot 82 | 88.75 | 17.75 | 0.01 | 0.005 | 0.04 | 148.617 | 2.56 | 257.72 |
| Plot 83 | 193.8 | 38.76 | 0.01 | 0.008 | 0.04 | 171.336 | 3.50 | 407.46 |
| Plot 84 | 217.09 | 43.42 | 0.01 | 0.005 | 0.04 | 159.714 | 1.59 | 421.87 |
| Plot 85 | 159.15 | 31.83 | 0.02 | 0.005 | 0.05 | 189.468 | 0.36 | 380.87 |
| Plot 86 | 180.3 | 36.06 | 0.01 | 0.006 | 0.03 | 148.8 | 4.75 | 369.96 |
| Plot 87 | 113.35 | 22.67 | 0.01 | 0.004 | 0.03 | 171.99 | 1.87 | 309.92 |
| Plot 88 | 121.16 | 24.23 | 0.02 | 0.007 | 0.02 | 185.442 | 0.85 | 331.74 |
| Plot 89 | 151.43 | 30.29 | 0.02 | 0.007 | 0.04 | 148.347 | 0.80 | 330.94 |
| Plot 90 | 217.58 | 43.52 | 0.02 | 0.005 | 0.02 | 200.07 | 2.55 | 463.76 |
