# Supplementary material for: Carbon stock of the various carbon pools in Gerba-Dima moist Afromontane forest, South-western Ethiopia
Source: Carbon Balance Manag. 2019 Feb 2;14:1. doi: 10.1186/s13021-019-0116-x (PMC6446976; doi:10.1186/s13021-019-0116-x)
Supplement: Supplementary file 2 — Additional file 2: Appendix S1. AGB, BGB, AGC, BGC and Carbon sequestered (CO2 equivalent) per tree of species in Gerba Dima Forest. [file 13021_2019_116_MOESM2_ESM.docx]

**Appendix S1. AGB, BGB, AGC, BGC and Carbon sequestered (CO_2_ equivalent) per tree of species in Gerba Dima Forest**

| **Species Name** | **No**  **of stems** | **Mean DBH** | **AGB ton ha^-1^ per single tree** | **BGB ton ha^-1^ per single tree** | **Total B ton ha^-1^ per single tree** | **AGC ton ha^-1^ per single tree** | **BGC ton ha^-1^ per single tree** | **Total C stock ton ha^-1^ per single tree** | **CO2 eqv.ton ha^-1^ per single tree** |
| --- | --- | --- | --- | --- | --- | --- | --- | --- | --- |
| *Ekebergia capensis* | 1.00 | 147.86 | 190.96 | 38.19 | 229.15 | 89.75 | 17.95 | 107.70 | 395.26 |
| *Schefflera abyssinica* | 293 | 123.90 | 85.74 | 17.15 | 102.89 | 40.30 | 8.06 | 48.36 | 177.48 |
| *Pouteria adolfi-friederici* | 23 | 58.72 | 63.03 | 12.61 | 75.64 | 29.62 | 5.92 | 35.54 | 130.45 |
| *Prunus Africana* | 31 | 48.47 | 44.75 | 8.95 | 53.70 | 21.03 | 4.21 | 25.24 | 92.62 |
| *Elaeodendron buchananii* | 47 | 35.67 | 42.81 | 8.56 | 51.37 | 20.12 | 4.02 | 24.14 | 88.61 |
| *Olea welwitschii* | 40 | 39.69 | 34.71 | 6.94 | 41.65 | 16.31 | 3.26 | 19.57 | 71.83 |
| *Sapium ellipticum* | 4 | 68.08 | 34.49 | 6.90 | 41.39 | 16.21 | 3.24 | 19.45 | 71.39 |
| *Trilepisium madagascariense* | 19 | 49.02 | 28.57 | 5.71 | 34.28 | 13.43 | 2.69 | 16.12 | 59.15 |
| *Polyscias fulva* | 31 | 58.31 | 21.57 | 4.31 | 25.88 | 10.14 | 2.03 | 12.17 | 44.66 |
| *Ficus sur* | 7 | 53.75 | 21.13 | 4.23 | 25.36 | 9.93 | 1.99 | 11.92 | 43.73 |
| *Ficus ovata* | 1.00 | 70.00 | 19.68 | 3.94 | 23.61 | 9.25 | 1.85 | 11.10 | 40.73 |
| *Celtis Africana* | 68 | 40.06 | 18.39 | 3.68 | 22.07 | 8.64 | 1.73 | 10.37 | 38.05 |
| *Bersama abyssinica* | 1.00 | 25.35 | 17.19 | 3.44 | 20.63 | 8.08 | 1.62 | 9.70 | 35.58 |
| *Syzygium guineense* | 5 | 31.82 | 16.45 | 3.29 | 19.74 | 7.73 | 1.55 | 9.28 | 34.04 |
| *Cledendron myricoides* | 29.00 | 42.00 | 15.78 | 3.16 | 18.93 | 7.42 | 1.48 | 8.90 | 32.66 |
| *Albizia gummifera* | 33 | 35.63 | 15.67 | 3.13 | 18.80 | 7.37 | 1.47 | 8.84 | 32.46 |
| *Albizia schimperiana* | 26.00 | 39.27 | 14.5 | 2.90 | 17.40 | 6.81 | 1.36 | 8.17 | 29.99 |
| *Ilex mitis* | 160 | 34.24 | 11.23 | 2.25 | 13.48 | 5.28 | 1.06 | 6.34 | 23.25 |
| *Croton macrostachyus* | 6.00 | 33.97 | 11.05 | 2.21 | 13.26 | 5.19 | 1.04 | 6.23 | 22.86 |
| *Millettia ferruginea* | 3 | 17.38 | 10.53 | 2.11 | 12.64 | 4.95 | 0.99 | 5.94 | 21.80 |
| *Brucea antidysenterica* | 78.00 | 9.38 | 9.15 | 1.83 | 10.98 | 4.3 | 0.86 | 5.16 | 18.94 |
| *Hallea rubrostipulata* | 5 | 27.12 | 8.63 | 1.73 | 10.36 | 4.06 | 0.81 | 4.87 | 17.88 |
| *Apodytes dimidiate* | 71.00 | 20.09 | 8.6 | 1.72 | 10.32 | 4.04 | 0.81 | 4.85 | 17.79 |
| *Cordia Africana* | 96 | 33.85 | 8.53 | 1.71 | 10.24 | 4.01 | 0.80 | 4.81 | 17.66 |
| *Cassipourea malosana* | 64.00 | 10.41 | 7.55 | 1.51 | 9.06 | 3.55 | 0.71 | 4.26 | 15.63 |
| *Macaranga capensis* | 7 | 28.92 | 7.48 | 1.50 | 8.98 | 3.51 | 0.70 | 4.21 | 15.46 |
| *Allophyllus abyssinicus* | 22.00 | 24.93 | 7.43 | 1.49 | 8.92 | 3.49 | 0.70 | 4.19 | 15.37 |
| *Trema orientalis* | 1.00 | 13.83 | 7.21 | 1.44 | 8.65 | 3.39 | 0.68 | 4.07 | 14.93 |
| *Trichilia dregeana* | 3.00 | 32.33 | 6.90 | 1.38 | 8.28 | 3.24 | 0.65 | 3.89 | 14.27 |
| *Ficus thonningi* | 1.00 | 16.00 | 6.48 | 1.30 | 7.78 | 3.05 | 0.61 | 3.66 | 13.43 |
| *Maesa lanceolata* | 105.00 | 12.70 | 6.30 | 1.26 | 7.56 | 2.96 | 0.59 | 3.55 | 13.04 |
| *Ritchiea albersii* | 2.00 | 9.68 | 5.96 | 1.19 | 7.15 | 2.80 | 0.56 | 3.36 | 12.33 |
| *Ehretia cymosa* | 4.00 | 13.14 | 4.38 | 0.88 | 5.26 | 2.06 | 0.41 | 2.47 | 9.07 |
| *Dracena steudneri* | 34.00 | 30.84 | 4.15 | 0.83 | 4.98 | 1.95 | 0.39 | 2.34 | 8.59 |
| *Lepidotrichilia volkensii* | 12.00 | 9.61 | 3.77 | 0.75 | 4.52 | 1.77 | 0.35 | 2.12 | 7.80 |
| *Dombeya torrid* | 14.00 | 12.42 | 3.36 | 0.67 | 4.03 | 1.58 | 0.32 | 1.90 | 6.96 |
| *Hippocratea pallens* | 7.00 | 5.82 | 3.27 | 0.65 | 3.92 | 1.54 | 0.31 | 1.85 | 6.78 |
| *Rothmannia urcelliformis* | 89.00 | 8.11 | 3.22 | 0.64 | 3.86 | 1.51 | 0.30 | 1.81 | 6.65 |
| *Vepris dainellii* | 94.00 | 7.96 | 3.21 | 0.64 | 3.85 | 1.51 | 0.30 | 1.81 | 6.65 |
| *Dracena afromontana* | 1.00 | 13.80 | 3.14 | 0.63 | 3.77 | 1.48 | 0.30 | 1.78 | 6.52 |
| *Chionanthus mildbraedii* | 66.00 | 9.00 | 3.11 | 0.62 | 3.73 | 1.46 | 0.29 | 1.75 | 6.44 |
| *Teclea nobilis* | 69.00 | 7.88 | 2.98 | 0.60 | 3.58 | 1.40 | 0.28 | 1.68 | 6.17 |
| *Combretum paniculatum* | 48.00 | 5.50 | 2.86 | 0.57 | 3.43 | 1.35 | 0.27 | 1.62 | 5.95 |
| *Vernonia amygdalina* | 4.00 | 9.60 | 2.86 | 0.57 | 3.43 | 1.35 | 0.27 | 1.62 | 5.95 |
| *Ensete ventericosum* | 56.00 | 50.00 | 2.85 | 0.57 | 3.42 | 1.34 | 0.27 | 1.61 | 5.90 |
| *Galiniera saxifrage* | 193.00 | 9.16 | 2.83 | 0.57 | 3.40 | 1.33 | 0.27 | 1.60 | 5.86 |
| *Canthium oligocarpum* | 80.00 | 10.75 | 2.81 | 0.56 | 3.37 | 1.32 | 0.26 | 1.58 | 5.81 |
| *Hippocratea Africana* | 38.00 | 5.61 | 2.76 | 0.55 | 3.31 | 1.30 | 0.26 | 1.56 | 5.73 |
| *Olea capensis* | 76.00 | 7.69 | 2.73 | 0.55 | 3.28 | 1.28 | 0.26 | 1.54 | 5.64 |
| *Pittosporum viridiflorum* | 8.00 | 7.12 | 2.56 | 0.51 | 3.07 | 1.20 | 0.24 | 1.44 | 5.28 |
| *Gouania longispicata* | 1.00 | 6.00 | 2.46 | 0.49 | 2.95 | 1.16 | 0.23 | 1.39 | 5.11 |
| *Oxyanthus speciosus* | 8.00 | 7.83 | 2.44 | 0.49 | 2.93 | 1.15 | 0.23 | 1.38 | 5.06 |
| *Alangium chinesis* | 44.00 | 23.00 | 2.38 | 0.48 | 2.86 | 1.12 | 0.22 | 1.34 | 4.94 |
| *Dalbergia lacteal* | 178.00 | 5.00 | 2.34 | 0.47 | 2.81 | 1.1 | 0.22 | 1.32 | 4.84 |
| *Cyathea manniana* | 32.00 | 11.50 | 1.98 | 0.40 | 2.38 | 0.93 | 0.19 | 1.12 | 4.10 |
| *Taccaza apiculata* | 8.00 | 5.50 | 1.74 | 0.35 | 2.09 | 0.82 | 0.16 | 0.98 | 3.61 |
| *Tiliacora troupinii* | 1.00 | 5.45 | 1.67 | 0.33 | 2.00 | 0.79 | 0.16 | 0.95 | 3.48 |
| *Landolphia buchananii* | 5.00 | 5.57 | 1.68 | 0.34 | 2.02 | 0.79 | 0.16 | 0.95 | 3.48 |
| *Jasminum abyssinicum* | 41.00 | 5.00 | 1.65 | 0.33 | 1.99 | 0.78 | 0.16 | 0.93 | 3.42 |
| *Seriostachys scandus* | 37.00 | 5.25 | 1.58 | 0.32 | 1.90 | 0.74 | 0.15 | 0.89 | 3.26 |
| *Clematis longicauda* | 73.00 | 5.00 | 1.37 | 0.27 | 1.64 | 0.64 | 0.13 | 0.77 | 2.82 |
| *Maytenus gracilipes* | 1.00 | 5.80 | 1.33 | 0.27 | 1.60 | 0.63 | 0.13 | 0.76 | 2.77 |
| *Erythrococca trichogyne* | 8.00 | 6.00 | 1.23 | 0.25 | 1.48 | 0.58 | 0.12 | 0.70 | 2.55 |
| *Phonix reclinata* | 1.00 | 22.25 | 1.22 | 0.24 | 1.46 | 0.57 | 0.11 | 0.68 | 2.51 |
| *Schefflera myriantha* | 3.00 | 5.00 | 1.16 | 0.23 | 1.39 | 0.54 | 0.11 | 0.65 | 2.39 |
| *Clausena anisata* | 109.00 | 6.38 | 1.11 | 0.22 | 1.33 | 0.52 | 0.10 | 0.62 | 2.29 |
| *Psychotria orophila* | 2.00 | 6.30 | 1.00 | 0.20 | 1.20 | 0.47 | 0.09 | 0.56 | 2.07 |
| *Pterolobium stellatum* | 29.00 | 3.80 | 0.97 | 0.19 | 1.16 | 0.45 | 0.09 | 0.55 | 2.00 |
| *Flacourtia indica* | 30.00 | 5.50 | 0.93 | 0.19 | 1.12 | 0.44 | 0.09 | 0.53 | 1.94 |
| *Solanaceo manni* | 4.00 | 20.00 | 0.89 | 0.18 | 1.07 | 0.42 | 0.08 | 0.50 | 1.85 |
| *Phyllanthus sepialis* | 3.00 | 5.00 | 0.71 | 0.14 | 0.86 | 0.34 | 0.07 | 0.40 | 1.48 |
| *Coffea arabica* | 17.00 | 5.33 | 0.68 | 0.14 | 0.82 | 0.32 | 0.06 | 0.38 | 1.41 |
| *Deinbollia kilimandscharica* | 181.00 | 5.05 | 0.54 | 0.11 | 0.65 | 0.26 | 0.05 | 0.31 | 1.15 |
| *Solanecio gigas* | 5.00 | 5.35 | 0.54 | 0.11 | 0.65 | 0.25 | 0.05 | 0.30 | 1.10 |
| *Vernonia rueppellii* | 1.00 | 5.00 | 0.35 | 0.07 | 0.42 | 0.17 | 0.03 | 0.20 | 0.73 |
