# Supplementary material for: Carbon stock of the various carbon pools in Gerba-Dima moist Afromontane forest, South-western Ethiopia
Source: Carbon Balance Manag. 2019 Feb 2;14:1. doi: 10.1186/s13021-019-0116-x (PMC6446976; doi:10.1186/s13021-019-0116-x)
Supplement: Supplementary file 3 — Additional file 3. Bulk Density, SOM, SOC and CO2 equivalent for each study plot in Gerba Dima Forest. [file 13021_2019_116_MOESM3_ESM.docx]

| **Plot number** | **Bulk Density (gcm^-3^)** | **Depth (cm)** | **% OM** | **% C** | **SOC(t ha^-1^)** | **CO2 equivalent(t ha^-1^)** |
| --- | --- | --- | --- | --- | --- | --- |
| 1 | 0.68 | 30 | 11.06 | 6.41 | 130.76 | 479.90 |
| 2 | 0.69 | 30 | 11.48 | 6.66 | 137.86 | 505.95 |
| 3 | 0.59 | 30 | 13.21 | 7.66 | 135.58 | 497.59 |
| 4 | 0.67 | 30 | 10.15 | 5.89 | 118.39 | 434.49 |
| 5 | 0.68 | 30 | 17.18 | 9.96 | 203.18 | 745.69 |
| 6 | 0.7 | 30 | 15.76 | 9.14 | 191.94 | 704.42 |
| 7 | 0.64 | 30 | 15.56 | 9.03 | 173.38 | 636.29 |
| 8 | 0.74 | 30 | 14.64 | 8.49 | 188.48 | 691.71 |
| 9 | 0.56 | 30 | 13.82 | 8.01 | 134.57 | 493.86 |
| 10 | 0.63 | 30 | 15.45 | 8.96 | 169.34 | 621.49 |
| 11 | 0.59 | 30 | 19.73 | 11.45 | 202.67 | 743.78 |
| 12 | 0.59 | 30 | 15.48 | 8.98 | 158.95 | 583.33 |
| 13 | 0.59 | 30 | 16.36 | 9.49 | 167.97 | 616.46 |
| 14 | 0.64 | 30 | 20 | 11.6 | 222.72 | 817.38 |
| 15 | 0.59 | 30 | 16.74 | 9.71 | 171.87 | 630.75 |
| 16 | 0.64 | 30 | 13.21 | 7.66 | 147.07 | 539.75 |
| 17 | 0.58 | 30 | 16.94 | 9.82 | 170.87 | 627.09 |
| 18 | 0.62 | 30 | 21.77 | 12.63 | 234.92 | 862.15 |
| 19 | 0.66 | 30 | 18.95 | 10.99 | 217.60 | 798.60 |
| 20 | 0.59 | 30 | 17.06 | 9.89 | 175.05 | 642.44 |
| 21 | 0.58 | 30 | 15.5 | 8.99 | 156.43 | 574.08 |
| 22 | 0.56 | 30 | 15.74 | 9.13 | 153.38 | 562.92 |
| 23 | 0.53 | 30 | 17.18 | 9.96 | 158.36 | 581.20 |
| 24 | 0.53 | 30 | 13.98 | 8.11 | 128.95 | 473.24 |
| 25 | 0.51 | 30 | 17.59 | 10.2 | 156.06 | 572.74 |
| 26 | 0.87 | 30 | 15.08 | 8.75 | 228.38 | 838.14 |
| 27 | 0.5 | 30 | 18.66 | 10.82 | 162.30 | 595.64 |
| 28 | 0.58 | 30 | 15.64 | 9.07 | 157.82 | 579.19 |
| 29 | 0.54 | 30 | 16.76 | 9.72 | 157.46 | 577.89 |
| 30 | 0.57 | 30 | 14.81 | 8.59 | 146.89 | 539.08 |
| 31 | 0.51 | 30 | 12.97 | 7.52 | 115.06 | 422.26 |
| 32 | 0.64 | 30 | 13.34 | 7.74 | 148.61 | 545.39 |
| 33 | 0.76 | 30 | 15.71 | 9.11 | 207.71 | 762.29 |
| 34 | 0.49 | 30 | 17.9 | 10.38 | 152.59 | 559.99 |
| 35 | 0.45 | 30 | 19.02 | 11.03 | 148.91 | 546.48 |
| 36 | 0.59 | 30 | 17.16 | 9.96 | 176.29 | 646.99 |
| 37 | 0.45 | 30 | 19.45 | 11.28 | 152.28 | 558.87 |
| 38 | 0.55 | 30 | 19.66 | 11.4 | 188.10 | 690.33 |
| 39 | 0.57 | 30 | 14.38 | 8.34 | 142.61 | 523.39 |
| 40 | 0.49 | 30 | 15.87 | 9.21 | 135.39 | 496.87 |
| 41 | 0.58 | 30 | 16.44 | 9.53 | 165.82 | 608.57 |
| 42 | 0.5 | 30 | 15.47 | 8.97 | 134.55 | 493.80 |
| 43 | 0.63 | 30 | 14.52 | 8.42 | 159.14 | 584.04 |
| 44 | 0.45 | 30 | 17.01 | 9.87 | 133.25 | 489.01 |
| 45 | 0.56 | 30 | 12.37 | 7.18 | 120.62 | 442.69 |
| 46 | 0.55 | 30 | 14.86 | 8.62 | 142.23 | 521.98 |
| 47 | 0.56 | 30 | 15.19 | 8.81 | 148.01 | 543.19 |
| 48 | 0.63 | 30 | 13.96 | 8.1 | 153.09 | 561.84 |
| 49 | 0.64 | 30 | 15.35 | 8.9 | 170.88 | 627.13 |
| 50 | 0.4 | 30 | 15.32 | 8.89 | 106.68 | 391.52 |
| 51 | 0.65 | 30 | 14.58 | 8.45 | 164.78 | 604.72 |
| 52 | 0.58 | 30 | 13.05 | 7.57 | 131.72 | 483.41 |
| 53 | 0.59 | 30 | 16.55 | 9.6 | 169.92 | 623.61 |
| 54 | 0.56 | 30 | 15.61 | 9.05 | 152.04 | 557.99 |
| 55 | 0.63 | 30 | 12.65 | 7.34 | 138.73 | 509.12 |
| 56 | 0.43 | 30 | 15.51 | 9 | 116.10 | 426.09 |
| 57 | 0.9 | 30 | 15.41 | 8.94 | 241.38 | 885.86 |
| 58 | 0.7 | 30 | 15.16 | 8.79 | 184.59 | 677.45 |
| 59 | 0.71 | 30 | 17.2 | 9.98 | 212.57 | 780.15 |
| 60 | 0.47 | 30 | 15.25 | 8.85 | 124.79 | 457.96 |
| 61 | 0.58 | 30 | 17.6 | 10.21 | 177.65 | 651.99 |
| 62 | 0.58 | 30 | 16.52 | 9.58 | 166.69 | 611.76 |
| 63 | 0.56 | 30 | 17.5 | 10.15 | 170.52 | 625.81 |
| 64 | 0.49 | 30 | 20.26 | 11.75 | 172.73 | 633.90 |
| 65 | 0.55 | 30 | 17.64 | 10.23 | 168.80 | 619.48 |
| 66 | 0.46 | 30 | 34.91 | 20.25 | 279.45 | 1025.58 |
| 67 | 0.55 | 30 | 13.17 | 7.64 | 126.06 | 462.64 |
| 68 | 0.5 | 30 | 17.1 | 9.92 | 148.80 | 546.10 |
| 69 | 0.58 | 30 | 17.29 | 10.03 | 174.52 | 640.50 |
| 70 | 0.49 | 30 | 16.58 | 9.62 | 141.41 | 518.99 |
| 71 | 0.48 | 30 | 15.81 | 9.17 | 132.05 | 484.62 |
| 72 | 0.51 | 30 | 15.78 | 9.15 | 140.00 | 513.78 |
| 73 | 0.58 | 30 | 14.08 | 8.17 | 142.16 | 521.72 |
| 74 | 0.66 | 30 | 12.21 | 7.08 | 140.18 | 514.48 |
| 75 | 0.6 | 30 | 15.61 | 9.05 | 162.90 | 597.84 |
| 76 | 0.62 | 30 | 16.87 | 9.78 | 181.91 | 667.60 |
| 77 | 0.62 | 30 | 17.18 | 9.96 | 185.26 | 679.89 |
| 78 | 0.54 | 30 | 18.21 | 10.56 | 171.07 | 627.83 |
| 79 | 0.53 | 30 | 17.03 | 9.88 | 157.09 | 576.53 |
| 80 | 0.52 | 30 | 16.89 | 9.8 | 152.88 | 561.07 |
| 81 | 0.47 | 30 | 14.89 | 8.64 | 121.82 | 447.09 |
| 82 | 0.49 | 30 | 17.44 | 10.11 | 148.62 | 545.42 |
| 83 | 0.59 | 30 | 16.7 | 9.68 | 171.34 | 628.80 |
| 84 | 0.57 | 30 | 16.1 | 9.34 | 159.71 | 586.15 |
| 85 | 0.57 | 30 | 19.1 | 11.08 | 189.47 | 695.35 |
| 86 | 0.5 | 30 | 17.1 | 9.92 | 148.80 | 546.10 |
| 87 | 0.63 | 30 | 15.7 | 9.1 | 171.99 | 631.20 |
| 88 | 0.62 | 30 | 17.19 | 9.97 | 185.44 | 680.57 |
| 89 | 0.53 | 30 | 16.09 | 9.33 | 148.35 | 544.43 |
| 90 | 0.65 | 30 | 17.69 | 10.26 | 200.07 | 734.26 |
